# Vision within the blind spot: a new test to quantify melanopsin pathway sensitivity

**DOI:** 10.1038/s41433-024-03102-4

**Published:** 2024-05-02

**Authors:** Jeff Rabin, Jewearly Lundby, Madison Brase, Ally Vu, Austin Millis, Grant Wood, Dorin Huq, Erica Poole

**Affiliations:** University of the Incarnate Word Rosenberg School of Optometry, San Antonio, TX USA

**Keywords:** Eye manifestations, Visual system

The physiological blind spot (BS) is the spatial projection of the optic nerve head wherein ganglion cell axons collect to form the optic nerve. The BS is 15.5° temporal to fixation, 5.5° wide by 7° high. Since the optic nerve head lacks photoreceptors, the BS is presumed to be an absolute scotoma readily mapped on tangent screen, but perceptually filled-in during normal viewing. Intrinsically photosensitive sensitive retinal ganglion cells (ipRGCs) absorb light with melanopsin mediating pupil responses, photoentrainment, cognition, alertness, and vision [1–4]. Since ipRGC axons contain melanopsin, they may respond to appropriate stimulation in the BS [4]. Our purpose was to: (1) determine whether vision can occur within the BS via ipRGCs (2) exploit this epiphenomenon to develop a rapid test of melanopsin pathway sensitivity.

**Fig. 1:**
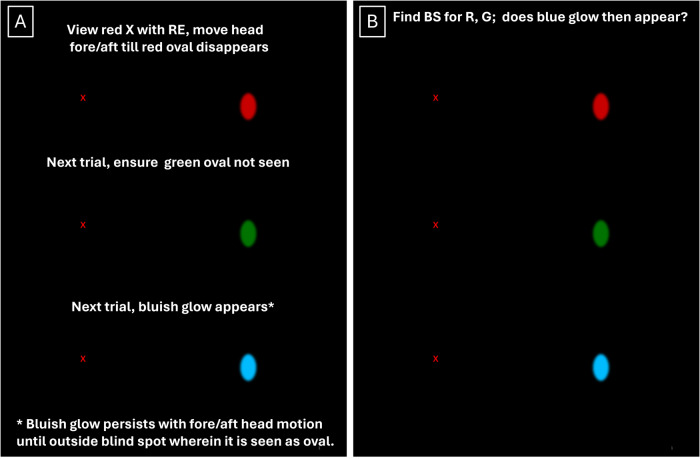
The Blind Spot Vision Test. **A** An extract from the Blind Spot Vision Test (BSVT) shown diagrammatically. With left eye occluded, the observer is seated before the display with right eye directly in front of the red X (top). The observer moves her/his head fore/aft until the red oval disappears and maintains the head at the approximate midpoint of the range of red oval disappearance. The next trial (middle) displays the green oval to ensure that it disappears as well. The next trial displays the ipRGC optimized blue oval and the observer reports if anything is seen and to describe its color. This was followed by the method of limits to determine the lowest blue luminance perceived (mean of three trials). **B** The reader may wish to experience disappearance of the red and green ovals followed by appearance of a less well-formed bluish glow or similar percept by following the steps described for Fig. 1A coupled with moving the head up and down or the computer image. It is recommended that this be conducted in a dark setting. Due to changes in chromaticity and luminance in the published images, this demonstration may not be optimal for the reader.

30 healthy adults provided written informed consent to participate in our IRB approved protocol (mean age±SD: 27 ± 9). Visual acuity, contrast sensitivity, and Ishihara testing verified normal vision and color vision. The Blind Spot Vision Test (BSVT, Fig. 1) was displayed on a Microsoft Surface in a dark room with left eye occluded. The subject’s right eye was centred before a red X (left side of black display viewed at 50–60 cm). The blind spot target was a 1.4° x 2.5° vertical oval 15° temporal to fixation. The first stimulus was red followed by a green oval each 12.7 cd/m^2^. The subject moved fore/aft until the red oval disappeared followed by green oval disappearance to ensure targets were in the BS. This blue oval (41.2 cd/m^2^, CIE *x,y* = 0.206, 0.278, dominant wavelength 480 nm, melanopsin peak) [1–3] was presented next. In response to the blue oval, subjects reported if anything appeared and its perceived color. The method of descending/ascending limits was used to determine three blue target thresholds by changing luminance up/down in 0.05 log steps. Mean, confidence intervals (CI), and the coefficient of repeatability (COR) were calculated from pooled data.

**Fig. 2:**
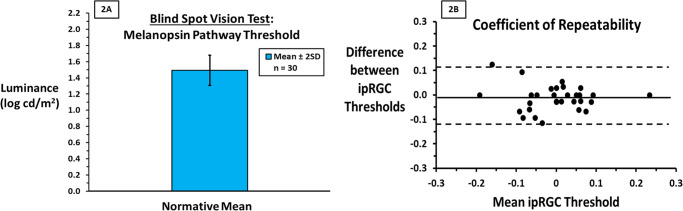
Normative Values for the Blind Spot Vision Test. **A** The mean ipRGC luminance detection threshold and 95% CI (±2 SD) is plotted (log value equivalent to 32 cd/m^2^) for 30 visually normal subjects to establish an initial normative database. Three subjects, tested in the same manner but in the nasal field where the BS is not present, showed red, green and blue thresholds >100X lower than the putative ipRGC thresholds in the BSVT consistent with much higher sensitivity of cone and/or rod photoreceptors (mediating temporal retina detection) compared to ipRGCs [1–5]. **B** Bland-Altman plot showing the pooled differences in ipRGC threshold for each subject’s right and left eye plotted against their means. The mean difference (−0.01 log cd/m^2^) is represented by the bold line and the dotted lines represent upper and lower limits of the COR (0.11 log cd/m^2^). Hence the COR represents the 95% confidence interval for within patient change; a difference exceeding 0.11 is considered significant. Like normative data, the COR is essential for serially monitoring of patients over time.

After BS determination (red, green oval disappearance) 100% reported reappearance of a light blue haze or glow in response to the blue oval. Two subjects tested at closer and farther distances reported that the glow became a well-formed peripheral blue oval suggesting cone mediation at distances outside the BS. Mean ± 2 SD ipRGC BSVT threshold was 32.0 ± 12.6 cd/m^2^ (1.5 ± 0.2 log cd/m^2^, Fig. 2A). The COR was 0.11 log cd/m^2^ (95% CI for within-subject change, Fig. 2B). Red, green, and blue thresholds from three subjects to stimuli 15° *nasal* to fixation were > 100X lower than BSVT thresholds substantiating mediation of the BSVT by less sensitive ipRGCs [1–4].

Study limitations include possible artifacts from fixational shifts or subject reliability which could be mitigated by fixation monitoring and catch trials. The results do support visual perception within the BS with stimuli optimized for ipRGCs. Testing across a blue luminance range yielded repeatable thresholds providing a metric of ipRGC pathway sensitivity. Potential applications include detection/monitoring of ocular, systemic and/or neurologic disease, and mood, alertness, pupil, and circadian rhythm function. BVST testing may also serve as an outcome measure for gene therapy efficacy in eye disease [5].
